# Overexpression of the Peach Transcription Factor Early Bud-Break 1 Leads to More Branches in Poplar

**DOI:** 10.3389/fpls.2021.681283

**Published:** 2021-06-17

**Authors:** Xuehui Zhao, Binbin Wen, Chen Li, Qiuping Tan, Li Liu, Xiude Chen, Ling Li, Xiling Fu

**Affiliations:** ^1^College of Horticulture Science and Engineering, Shandong Agricultural University, Tai’an, China; ^2^State Key Laboratory of Crop Biology, Shandong Agricultural University, Tai’an, China; ^3^Shandong Collaborative Innovation Center for Fruit and Vegetable Production With High Quality and Efficiency, Tai’an, China; ^4^Shandong Academy of Grape, Shandong Academy of Agricultural Sciences, Jinan, China

**Keywords:** PpEBB1, shoot branching, sugars, amino acids, brassinosteroids, light response

## Abstract

Shoot branching is an important adaptive trait that determines plant architecture. In a previous study, the *Early bud-break 1* (*EBB1*) gene in peach (*Prunus persica* var. *nectarina*) cultivar Zhongyou 4 was transformed into poplar (*Populus trichocarpa*). *PpEBB1-oe* poplar showed a more branched phenotype. To understand the potential mechanisms underlying the EBB1-mediated branching, transcriptomic and proteomics analyses were used. The results showed that a large number of differentially expressed genes (DEGs)/differentially expressed proteins (DEPs) associated with light response, sugars, brassinosteroids (BR), and nitrogen metabolism were significantly enriched in *PpEBB1-oe* poplar. In addition, contents of sugars, BR, and amino acids were measured. Results showed that PpEBB1 significantly promoted the accumulation of fructose, glucose, sucrose, trehalose, and starch. Contents of brassinolide (BL), castasterone (CS), and 6-deoxocathasterone (6-deoxoCS) were all significantly changed with overexpressing *PpEBB1*. Various types of amino acids were measured and four of them were significantly improved in *PpEBB1-oe* poplar, including aspartic acid (Asp), arginine (Arg), cysteine (Cys), and tryptohpan (Trp). Taken together, shoot branching is a process controlled by a complex regulatory network, and PpEBB1 may play important roles in this process through the coordinating multiple metabolic pathways involved in shoot branching, including light response, phytohormones, sugars, and nitrogen.

## Introduction

Early bud-break 1 (EBB1), an ethylene-responsive factor (ERF), which is a transcription factor, is the putative orthologue of dornröschen/dornröschen-like (DRN/L); it is also known as enhancer of shoot regeneration1/2 (ESR1/2) in Arabidopsis (Chandler, 2018). Since overexpression of *EBB1* promoted bud break, whereas knockdown of *EBB1* delayed bud break, it was named EBB1, and this function was conserved in many deciduous woody trees, such as poplar, pear, and peach (Yordanov et al., 2014; Anh Tuan et al., 2016; Zhao et al., 2020). In Arabidopsis, DRN/L participates in many developmental processes, including embryogenesis, cotyledon organogenesis, and floral organogenesis (Chandler et al., 2007, 2008, 2011a,b; Cole et al., 2009; Chandler, 2018). *ESR1/2* can regulate *in vitro* shoot regeneration; overexpression of *ESR1* greatly increased the efficiency of shoot regeneration in root explants, while the *esr1 esr2* double mutation showed drastically decreased shoot regeneration efficiencies (Banno et al., 2001; Matsuo and Banno, 2008; Matsuo et al., 2011). Similarly, overexpression of EBB1 in poplar caused spontaneous regeneration from leaf disks and cambium-derived callus, resulting in an increased proliferation of sylleptic branches (Yordanov et al., 2014).

Shoot branching determines plant architecture, a plastic adaptive trait important for competing for light. Branches were formed from bud outgrowth and subsequent elongation of axillary buds in leaf axils. Shoot branching is regulated by both endogenous and environmental signals, including light, nutrition, hormones, and key genes (Drummond et al., 2015; Barbier et al., 2019). A lasting model suggests that auxin in the main stem competitively inhibits auxin export from the axillary buds, which is seen as a prerequisite for bud outgrowth. But some studies suggested that auxin export was important for young branch growth but not essential to trigger initiation of bud growth, whereas cytokinin and strigolactone have been identified as early regulators of bud outgrowth, although the role was not fully understood (Dun et al., 2012; Barbier et al., 2019; Chabikwa et al., 2019). Sugars play crucial roles in promoting shoot branching function both as signals and as sources of carbon (Patrick et al., 2013; Barbier et al., 2019). Nitrogen and phosphorus supply leads to more branches in petunia (*Petunia hybrida*; Drummond et al., 2015) and *Arabidopsis* (de Jong et al., 2014).

In a previous study (Zhao et al., 2020), the *EBB1* gene in peach (*Prunus persica* var. *nectarina*) cultivar Zhongyou 4 has been identified and overexpressed in poplar (*Populus trichocarpa*). In addition to promoting bud break, EBB1-overexpressing poplar had more branches. To explore the potential mechanisms underlying EBB1-mediated branching, we conducted transcriptomic and proteomics analyses using *PpEBB1-overexpressing* (*PpEBB1-oe*) and wild-type (WT) poplars.

## Materials and Methods

### Plant Material

A peach gene, *PpEBB1* has been transformed into poplar as described in a previous study (Zhao et al., 2020). The WT and transgenic poplars were grown in an aseptic Murashige and Skoog (MS) medium containing 0.1 mg·l^−1^ naphthyl acetic acid (NAA) under a 14:10 h day and (200 μmol·m^−2^·s^−1^) night light regimes at temperatures of 25 and 18°C, respectively. The stems were cut into several segments (about 1 cm) for subculture, each segment containing at least two axillary buds. The 6-week seedlings were used in this study. The axillary shoots of transgenic poplar and WT poplar were used for RNA sequencing (RNA-seq) analysis and protein extraction. The basal portions of the shoots were used for sugar content determination, brassinosteroid (BR) content determination, and individual amino acid determination.

### Total RNA Extraction and Quantitative Real-Time PCR

Total RNA was isolated using the RNA prep Pure Plant Kit (polysaccharide- and polyphenolic-rich; TIANGEN, Beijing, China) with three biological replicates. A NanoPhotometer P360 (Implen, Munich, Germany) was used to assess the quality and quantity of RNA. The PrimeScript RT reagent kit and gDNA Eraser (Perfect Real Time; Takara Biotechnology, Dalian, China) were used for reverse transcription. SYBR Premix Ex Taq (Takara Biotechnology, Dalian, China) was used for qRT-PCR. Primers are listed in Supplementary Table S1.

### RNA-Seq Analysis

RNA was isolated using the RNA prep Pure Plant Kit (polysaccharide- and polyphenolic-rich; Tiangen, Beijing, China). The concentration and purity were assessed by NanoPhotometer® spectrophotometer (Implen, CA, United States), Qubit®3.0 Fluorometer (Life Technologies, CA, United States), and Agilent 2100 RNA Nano 6,000 Assay Kit (Agilent Technologies, CA, United States). Oligo (dT) magnetic beads were used for mRNA enrichment and purification. The messenger RNA (mRNA) was fragmented by adding the fragmentation buffer. Then, the first strand of complementary DNA (cDNA) was synthesized using 6-base random primers with mRNA segments as the template. The second strand of cDNA was synthesized by adding buffer, dNTPs, RNaseH, and DNA Polymerase I. QIAQuick PCR kit was used for purification using VAHTS® Universal V6 RNA-seq Library Prep Kit for Illumina (Vazyme, Nanjing, China). Then the double-stranded cDNA was treated with terminal repair, base A, and a sequencing connector. Then the 450–500 bp fragment was recovered by agar-gel electrophoresis and amplified by PCR, to complete the preparation of the library. The prepared library was sequenced using the PE150 method in the Illumina platform. The clean data was obtained by removing low-quality sequences and joint contamination. Reads Count for each gene in each sample was counted by HTSeq v0.6.0, and fragments per kilobase million mapped reads (FPKM) were then calculated to estimate the expression level of genes in each sample. *P. trichocarpa* genome annotations were downloaded from the JGI database.^1^ DESeq2 v1.6.3 was used to identify DEGs between WT and transgenic poplar. Genes with *q*≤ 0.05 and |log2_ratio| ≥ 1 were identified as DEGs. The Gene Ontology (GO; http://geneontology.org/) enrichment of DEGs was determined by the hypergeometric test, in which the *p*-value was calculated and adjusted as the q-value, and the background data was genes in the whole genome. GO terms with *q* < 0.05 were considered to be significantly enriched. The Kyoto Encyclopedia of Genes and Genomes (KEGG) enrichment of DEGs was determined by the hypergeometric test, in which the *p*-value was adjusted by multiple comparisons as the q-value. KEGG terms with *q* < 0.05 were considered to be significantly enriched.

### Protein Extraction

Total protein was extracted with three biological replicates. Samples were collected and frozen in liquid nitrogen immediately. Then, the powder was transferred into a 5 ml centrifuge tube with lysis buffer (containing 1% Triton X-100, 10 mm dithiothreitol, 1% protease inhibitor cocktail, 50 μm PR-619, 3 μm TSA, 50 mm NAM, and 2 mm EDTA; v:v = 1:4) and sonicated three times on ice using a high-intensity ultrasonic processor (Scientz Biotechnology, Ningbo, China). An equal volume of Tris-saturated phenol (pH 8.0) was added, and the mixture was vortexed for 5 min. The upper phenol phase was transferred to a new centrifuge tube after centrifugation at 5,000 *g* at 4°C for 10 min, and five volumes of ammonium sulfate-saturated methanol were added and incubated at −20°C for at least 6 h to precipitate proteins. The supernatant was removed after centrifugation at 4°C for 10 min, and the precipitate was washed once with ice-cold methanol, followed by ice-cold acetone three times. Finally, 8 m urea was used to redissolve the protein, and the protein concentration was determined using a BCA protein assay kit (Beyotime, Nanjing, China) according to the instructions of the manufacturer.

### Trypsin Digestion

For digestion, the protein solution was reduced with 5 mm dithiothreitol for 30 min at 56°C and alkylated with 11 mm iodoacetamide for 15 min at room temperature in darkness. The protein sample was then diluted by adding 100 mm triethylammonium bicarbonate (TEAB) to urea concentrations less than 2 min. Finally, trypsin was added at a 1:50 trypsin-to-protein mass ratio for the first digestion overnight and a 1:100 trypsin-to-protein mass ratio for a second 4 h digestion.

### Tandem Mass Tag Labeling

After trypsin digestion, the peptide was desalted by a Strata X C18 SPE column (Phenomenex, Los Angeles, United States) and vacuum-dried. The peptide was reconstituted in 0.5 M TEAB and processed according to the protocol of the manufacturer for the tandem mass tag (TMT) kit. Briefly, one unit of TMT reagent was thawed and reconstituted in acetonitrile. The peptide mixtures were then incubated for 2 h at room temperature and pooled, desalted, and dried by vacuum centrifugation.

### High-Performance Liquid Chromatography Fractionation

The tryptic peptides were fractionated by high pH reverse-phase HPLC using an Agilent 300Extend C18 column (5 μm particles, 4.6 mm ID, 250 mm length). Briefly, peptides were first separated with a gradient of 8–32% acetonitrile (pH 9.0) over 60 min into 60 fractions. Then, the peptides were combined into 18 fractions and dried by vacuum centrifugation.

### Liquid Chromatography With Tandem Mass Spectrometry Analysis

The tryptic peptides were dissolved in 0.1% formic acid (solvent A) and directly loaded onto a homemade reversed-phase analytical column (15 cm length, 75 μm i.d.). The gradient was comprised of an increase from 6 to 23% solvent B (0.1% formic acid in 98% acetonitrile) over 26 min, from 23 to 35% in 8 min and increasing to 80% in 3 min, and then holding at 80% for the last 3 min, all at a constant flow rate of 400 nl/min on an EASY-nLC 1000 UPLC system.

The peptides were subjected to nanospray ionization (NSI) source followed by tandem mass spectrometry (MS/MS) in Q ExactiveTM Plus (Thermo Fisher Scientific, MA, United States) coupled online to the ultra-performance liquid chromatography (UPLC). The electrospray voltage applied was 2.0 kV. The m/z scan range was from 350 to 1,800 for a full scan, and intact peptides were detected in the Orbitrap at a resolution of 70,000. Peptides were then selected for MS/MS using the NCE setting of 28, and the fragments were detected in the Orbitrap at a resolution of 17,500. A data-dependent procedure alternated between one MS scan followed by 20 MS/MS scans with 15 s dynamic exclusions. Automatic gain control (AGC) was set at 5E4. The fixed first mass was set as 100 m/z.

### BR Measurements

Samples (1 g) of lyophilized liquid nitrogen were ground into a powder and extracted for 2 h at 4°C in 10 ml of 80% methanol. The extracts were centrifuged at 10,000 r/min for 5 min at 4°C. The supernatant was added to a Bond Elut column and eluted with 3 ml methanol. Then, the eluant was added into a Strata-X column and eluted with 3 ml methanol. Nitrogen flow was used to dry eluant and 200 μl methanol was added to redissolve. The sample was filtered through a 0.22 μm syringe for HPLC-MS/MS detection.

The extracted samples were quantified using a ZORBAX SB-C18 reversed-phase column (150 mm × 2.1 mm, 3.5 μm). Five microlitres of the sample were injected at a flow rate of 0.35 ml·min^−1^ and a column temperature of 35°C.

## Sugar Measurement

Sample (0.25 g) was pulverized with 2 ml 80% alcohol in a mortar and ultrasonically extracted for 60 min. The supernatant was collected at 8,000 *g* for 10 min. The extraction was repeated once. Precipitation was used for the determination of starch content according to a previous study (Fichtner et al., 2017). The upper portion was concentrated to 0.3 ml using nitrogen flow and extracted with 0.5 ml petroleum ether 3 times. The aqueous phase was collected for the determination of soluble sugars. Glucose, sucrose, trehalose, and fructose were measured using a chromatographic column (300 mm × 7.8 mm, 10 μm) and a Water 1,525 HPLC system. About 10 μl of the sample was injected at a flow rate of 0.4 ml·min^−1^ and at 80°C column temperature.

### Individual Amino Acid Measurements

Individual amino acids were measured according to the method used in the previous study (Gayen et al., 2016). Sample power (0.5 g) was placed in 10 ml of 6 M HCl containing 0.1% phenol at 110°C for approximately 20 h. The digested sample was filtered through a 0.22 μm syringe filter. Two hundred microlitres of the clear extract were taken in a 2 ml microcentrifuge tube along with 20 μl of n-leucine internal standard solution; then, 100 μl of triethylamine acetonitrile and 100 μl of phenyl isothiocyanate acetonitrile were added into the tube in turn. The sample was mixed well and allowed to stand at 25°C for 1 h. A sample of 10 μl was injected into an Amethyst C18-H column (250 mm × 4.6 mm, 5 μm) using a RIGOL L3000 HPLC at a flow rate of 0.4 ml·min^−1^, 40°C column temperature, and detected by a UV detector at a wavelength of 254 nm.

### Statistical Analysis

GraphPad Prism software was used to construct charts. Statistical analysis was performed using IBM SPSS Statistics 19 software to analyze the significance of differences among data, with a significance level of *p* < 0.05 under Duncan’s test.

## Results

### Overexpression of *PpEBB1* Leads to More Branches

**Figure 1:**
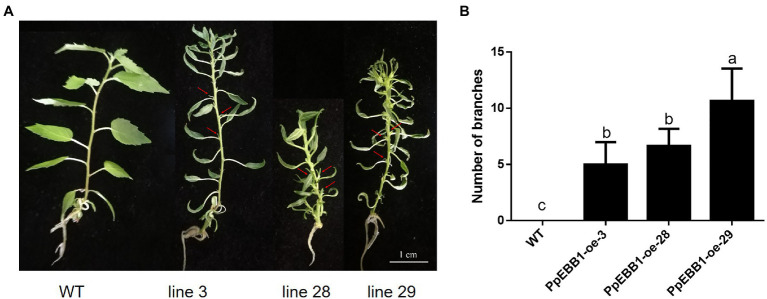
*PpEBB1-oe* poplars have more branches. **(A)** The axillary buds in *PpEBB1-oe* poplar lines are outgrowth, which are pointed out with arrows, whereas axillary buds in WT remain dormant. The scale in the figure is 1 cm. **(B)** Branches of *PpEBB1-oe* poplars are grown out in subculture, and there are more branches in *PpEBB1-oe* poplar lines compared with WT. Shoots (>1 mm) were counted. The values represent the means ± SDs of three replicates. Letters indicate significant differences by ANOVA followed by Duncan’s test (*p* < 0.05).

*PpEBB1-oe* poplars have been obtained from a previous study (Zhao et al., 2020). WT and *PpEBB1-oe* plantlets were cultured in an aseptic MS medium for long-days photoperiod for about 6 weeks. In *PpEBB1-oe* poplar, some axillary buds in the axils of leaves were released to grow out while the axillary buds of WT were inhibited (Figure 1A). As a result, overexpressing *EBB1* led to more syllectic branches compared with WT (Figure 1B; Supplementary Figure S1). Meanwhile, overexpression of *PpEBB1* led to smaller and curly leaves, which was also observed by Yordanov et al. (2014).

### Transcriptomic and Proteomic Changes in *PpEBB1-oe* Plants

To identify the molecular mechanisms by which PpEBB1 promotes branching, line 29, which has the highest *PpEBB1* expression level among all of the transgenic lines (Zhao et al., 2020), was used for transcriptome and tandem mass tag (TMT) proteome analysis. Significantly differentially expressed genes (DEGs) were identified based on their |log2Ratio| ≥ 1 and *q* < 0.05 threshold between the *PpEBB1-oe* line 29 and WT poplar. DEGs were used for GO term analysis and KEGG enrichment analysis. Results showed that a large number of DEGs were involved in light response, sugar metabolism, BR metabolism, plant hormone signal transduction, and photosynthesis (Datasets S1 and S2). The RNA-seq data were reliably validated by qRT-PCR and principal component analysis (PCA; Supplementary Figures S2, S3). Proteins with significant (*p* < 0.05) > 1.3 or <1/1.3-fold changes in relative abundance between *PpEBB1-oe* line 29 and WT were identified as differentially expressed proteins (DEPs). The result of PCA verified the repeatability and reliability of the proteomics data (Supplementary Figure S4). These DEPs were categorized into numerous metabolic processes by GO and KEGG analysis. Results showed that a large number of DEPs were associated with sugar metabolism, nitrogen metabolism, and amino acid metabolism (Datasets S3 and S4).

### GO Terms Related to Light Response

Light is a key environmental factor for bud outgrowth. Light quality and light intensity have a great influence on regulating branching. In this study, a large number of DEGs (Dataset S5) were enriched in light-associated GO terms, including “response to light stimulus” (GO:0009416), the response to light qualities (blue light, GO:0009637; red light, GO:0010114; and far-red light, GO:0010218), and light intensity (GO:0009642; GO:0009644; Dataset S1). Hence, these results indicate that EBB1 may influence the response of a plant to light, which may be one of the factors by which EBB1 promotes branching.

### DEGs and DEPs Related to Sugar Metabolism

**Figure 2:**
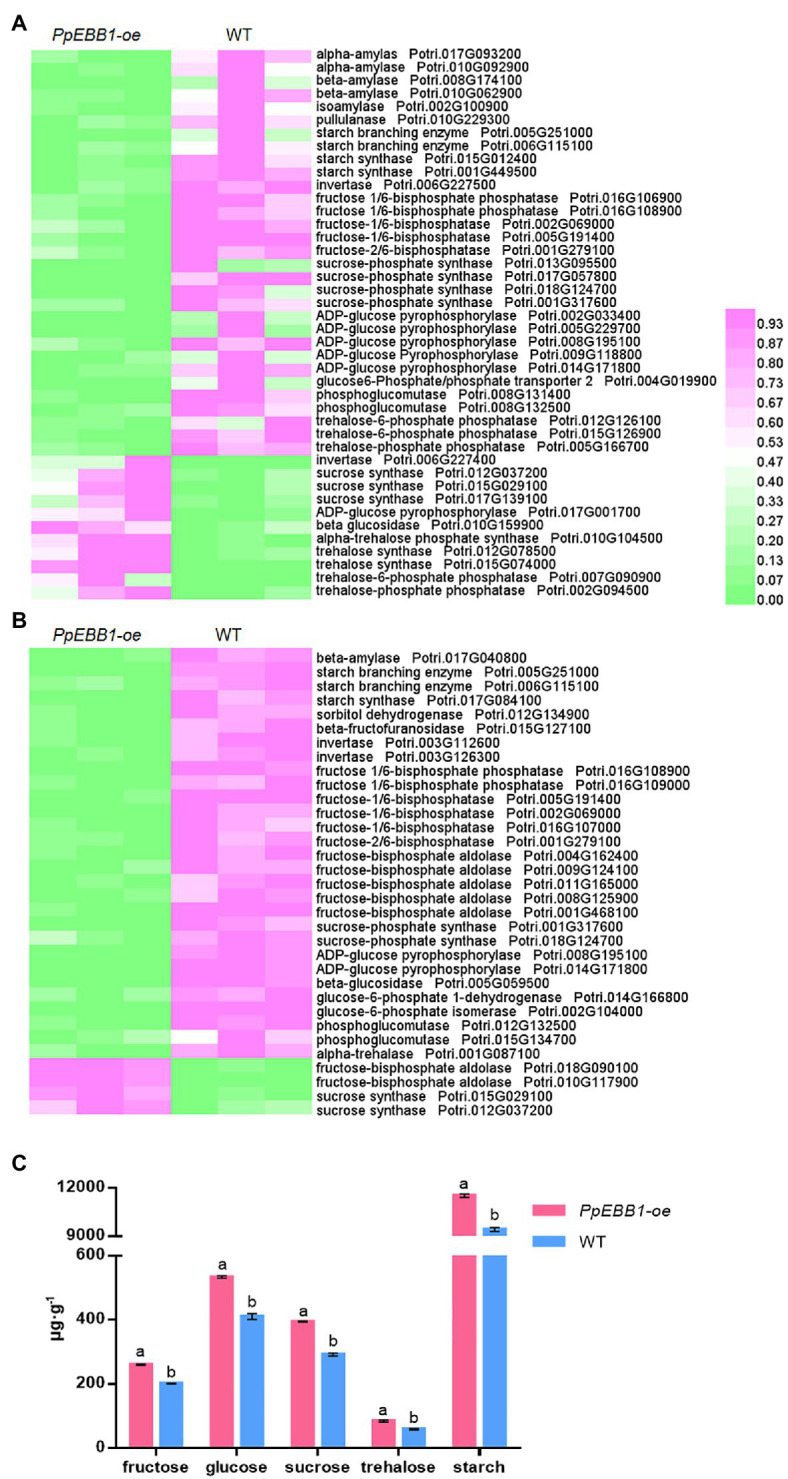
PpEBB1 greatly influenced sugar metabolism. **(A)** Differentially expressed genes (DEGs) involved in sugar metabolism between *PpEBB1-oe* and wild type (WT). Genes with significant |log2_ratio| ≥ 1 changes between *PpEBB1-oe* line 29 and WT were identified as DEGs. Purple represents higher expression, while green represents lower expression. **(B)** Differentially expressed proteins (DEPs) involved in sugar metabolism between *PpEBB1-oe* and WT. Proteins with significant (*p* < 0.05) > 1.3 or <1/1.3-fold changes between *PpEBB1-oe* line 29 and WT were identified as DEPs. Purple represents higher expression, while green represents lower expression. The expression value of DEGs and DEPs in transcriptomic and proteomics data were processed by Min-MaxNormalization from 0 to 1, and the scale is represented as the expression level. **(C)** Contents of different types of sugars in *PpEBB1-oe* and WT. The values represent the means ± SDs of three replicates. Letters indicate significant differences by ANOVA followed by Duncan’s test (*p* < 0.05).

The positive function of sugars on bud outgrowth has been well studied (Mason et al., 2014; Barbier et al., 2015a). Notably, from the transcriptomic data in the study, some GO terms related to sugar metabolism were significantly enriched (Dataset S1), including starch catabolic (GO:0005983) and metabolic (GO:0005982) processes, “response to sucrose” (GO:0009744), “hexose metabolic process” (GO:0019318), “fructose metabolic process” (GO:0006000), and “sucrose synthase activity” (GO:0016157). In addition, a KEGG pathway “starch and sucrose metabolism” (map00500) was identified (Dataset S2). DEGs involved in these sugar metabolism processes were selected and searched for the orthologs in Arabidopsis for detailed annotation (Dataset S6). A heatmap was formed using these DEGs (Figure 2A), and from Figure 2A, we can see that most of the DEGs involved in sugar metabolism are downregulated.

In addition, different sugar metabolism processes, including “fructose 1,6-bisphosphate 1-phosphatase activity” (GO:0042132), “sugar-phosphatase activity” (GO:0050308), “hexose biosynthetic process” (GO:0019319), “glucose metabolic process” (GO:0006006), “fructose metabolic process” (GO:0006000), “fructose and mannose metabolism” (pop00051), and “starch and sucrose metabolism” (pop00500), were significantly influenced, as verified by both the GO (Dataset S3) and KEGG (Dataset S4) enrichment analyses of the DEPs performed in this study. Most of these DEPs were downregulated in the *PpEBB1-oe* poplar (Figure 2B; Dataset S7), which was consistent with the transcriptomic results. These results indicate that PpEBB1 could have a great influence on sugar metabolism so that the levels of various types of sugar were measured to exactly understand the function of PpEBB1 on sugars. As shown in Figure 2C, the contents of different types of sugars detected in this study, including fructose, glucose, sucrose, trehalose, and starch, were all significantly increased in the *PpEBB1-oe* poplar. Due to a decrease in the expression of metabolism genes and proteins, the rates of catabolism or utilization may be affected resulting in an increase in *PpEBB1-oe* poplars.

### DEGs Related to BR Biosynthesis, Metabolism, and Signaling

**Figure 3:**
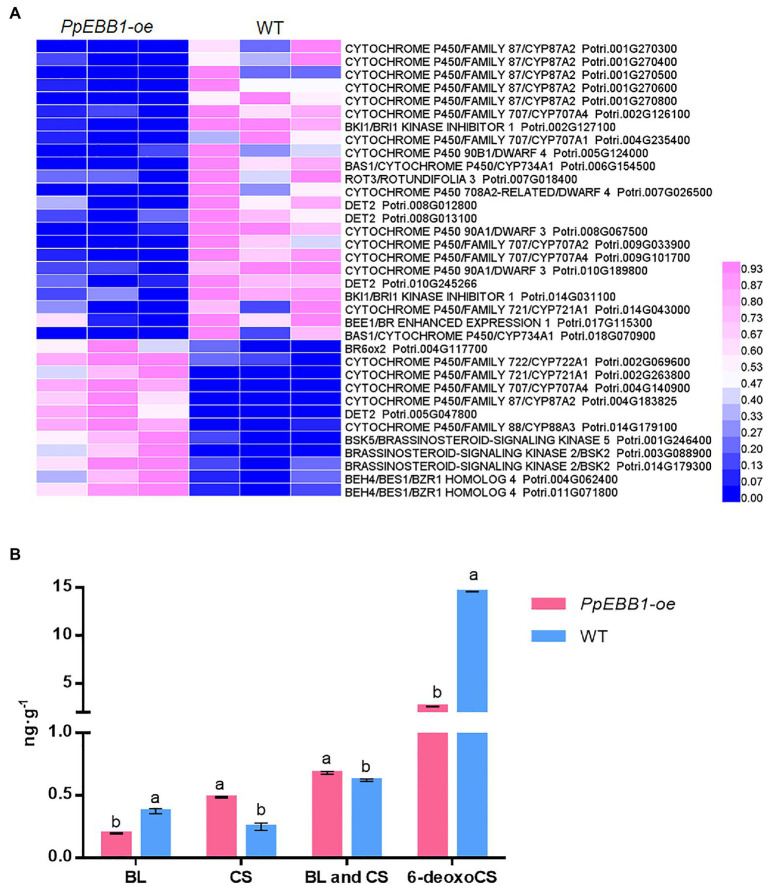
PpEBB1 greatly influenced brassinosteroid (BR) metabolism. **(A)** DEGs involved in BR biosynthesis, metabolism, and signaling between *PpEBB1-oe* and WT. Genes with significant |log2_ratio| ≥ 1 changes between *PpEBB1-oe* line 29 and WT were identified as DEGs. Purple represents higher expression, while blue represents lower expression. The expression value of DEGs in transcriptomic data were processed by Min-MaxNormalization from 0 to 1, and the scale is represented in the expression level. **(B)** Contents of BL, CS, and 6-deoxoCS in *PpEBB1-oe* and WT. The values represent the means ± SDs of three replicates. Letters indicate significant differences by ANOVA followed by Duncan’s test (*p* < 0.05).

GO (Dataset S1) and KEGG terms (Dataset S2) related to BR were identified, and they included “brassinosteroid biosynthetic process” (GO:0016132), “brassinosteroid metabolic process” (GO:0016131), “brassinosteroid homeostasis” (GO:0010268), “regulation of brassinosteroid biosynthetic process” (GO:0010422), and “brassinosteroid biosynthesis” (map00905). The expression levels of genes involved in the biosynthesis of BR (Supplementary Figure S5) including *DWARF* (*DWF*) *4*, *DWF6/de-etiolated 2* (*DET2*), *rotundifolia 3* (*ROT3*)/*CYP90C1*, *constitutive photomorphogenesis and dwarfism* (*CPD*)/*CTY90A1*, and *BR6ox1* were changed (Figure 3A; Dataset S8). *BAS1* is a BR-inactivating gene that converts castasterone (CS) and brassinolide (BL) to their C26 hydroxylated derivatives. Two differentially expressed *BAS1* genes in the *PpEBB1-oe* poplar were both downregulated (Figure 3A; Dataset S8). BKI1, interacting with brassinosteroid insensitive1 (BRI1) and negatively regulating BRI1 signaling (Wang and Chory, 2006; Wang et al., 2014), were downregulated, whereas *BSK* and *BES1/BZR1* genes were upregulated in *PpEBB1-oe* poplar.

The contents of BL, CS, and 6-deoxocathasterone (6-deoxoCS) were measured (Figure 3B). The 6-deoxoCS level was significantly decreased in *PpEBB1-oe* poplar, which could be transformed into CS under the action of BR6ox1/2 in the campestanol (CN)-independent pathway. CS, the immediate precursor of BL, was significantly increased in *PpEBB1-oe* poplar, while BL was significantly decreased. The total contents of CS and BL were compared between *PpEBB1-oe* poplar and WT because only CS and BL possess detectable biological activity (Yokota et al., 1982; Zhao and Li, 2012), and the results showed that the content of active BRs in *PpEBB1-oe* poplar was slightly higher than that in WT.

### Overexpression of EBB1 Altered Nitrogen Metabolism-Related Proteins and Amino Acid Content

**Figure 4:**
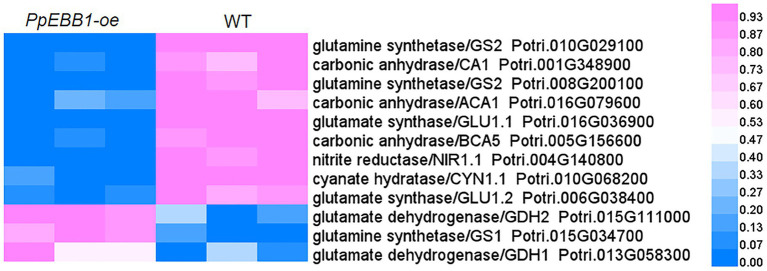
PpEBB1 greatly influenced nitrogen metabolism. DEPs involved in nitrogen metabolism between *PpEBB1-oe* and WT. Proteins with significant (*p* < 0.05) > 1.3 or <1/1.3-fold changes between *PpEBB1-oe* line 29 and WT were identified as DEPs. Purple represents higher expression, while blue represents lower expression. The expression value of DEPs in proteomics data were processed by Min-MaxNormalization from 0 to 1, and the scale is represented in the expression level.

**Figure 5:**
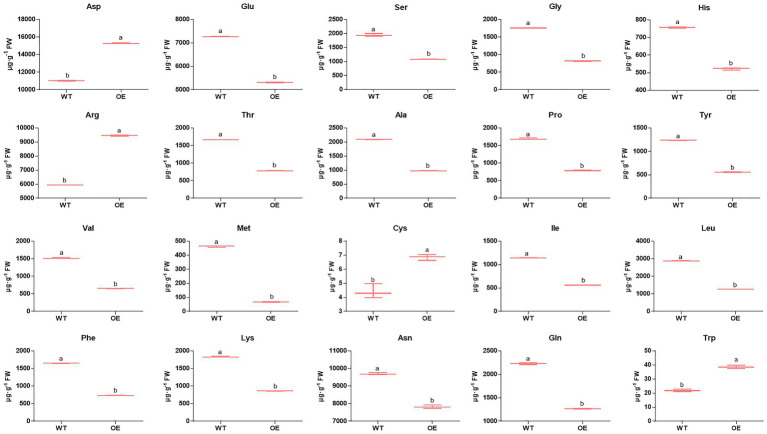
Overexpression of PpEBB1 made great influence on amino acid content. Twenty types of amino acid content are found in the basal portions of the shoots in WT and *PpEBB1-oe* poplar. The values represent the means ± SDs of three replicates. Letters indicate significant differences by ANOVA followed by Duncan’s test (*p* < 0.05).

Nitrogen is one of the most important mineral nutrients for plant growth and development, including regulating shoot branching (Drummond et al., 2015). Twelve DEPs were mapped in the nitrogen metabolism pathway (pop00910; Dataset S4) by KEGG analysis. Among these DEPs (Figure 4; Dataset S9), one nitrite reductase and two glutamate synthase proteins were downregulated, while two glutamate dehydrogenase proteins were upregulated, and two of the glutamine synthetase proteins were downregulated; the other was upregulated in *PpEBB1-oe* poplars. Nitrogen is partially converted to amino acids after assimilation by plants, and studies (Fichtner et al., 2017; Lu et al., 2018) found that there is a relationship between amino acids and shoot branching. GO and KEGG analysis identified some terms (Datasets S3 and S4) related to amino acids, including “aspartate family amino acid biosynthetic process” (GO:0009067), “cysteine and methionine metabolism” (pop00270), “histidine metabolism” (pop00340), “glycine, serine, and threonine metabolism” (pop00260), “tryptophan metabolism” (pop00380), “phenylalanine, tyrosine, and tryptophan biosynthesis” (pop00400), “arginine biosynthesis” (map00220), “alanine, aspartate, and glutamate metabolism” (map00250), and “biosynthesis of amino acids” (map01230). Hence, the content of 20 amino acids in *PpEBB1-oe* and WT poplars was determined. Compared with WT, *PpEBB1-oe* poplar only had higher levels of aspartic acid (Asp), arginine (Arg), cysteine (Cys), and tryptophan (Trp), whereas other amino acids were all significantly decreased (Figure 5).

## Discussion

It has been considered that EBB1 defined a conserved mechanism for the control of bud-break in woody perennials (Busov et al., 2016). In this study, we found that *PpEBB1-oe* poplars showed a more branched phenotype, which was also proposed by Yordanov et al. (2014). Shoot branching determines the plant architecture regulated by a complex network involving environmental signals, phytohormones, nutrition, some key genes, and the cross-talk function among these factors (Wang et al., 2018; Barbier et al., 2019). Light is a necessary photomorphogenic signal for triggering bud outgrowth (Roman et al., 2016). In this study results, six GO terms associated with the response to light were significantly enriched, including two responses to light intensity, and three responses to light quality, including blue, red, and far-red light (Dataset S1), suggesting that EBB1 is important for the response to different light signals. The effects of light intensity and light quality controlling branching have been well studied and hence, low light intensity and low red/far-red (R:FR) ratios inhibit bud outgrowth, leading to fewer branches (Demotes-Mainard et al., 2013; Furet et al., 2014; Drummond et al., 2015). Although the effect of blue light on branching was little studied in perennials, considering its direct effect on controlling stomata opening, photosynthesis, and sugar content (Muneer et al., 2014), it might be a potential factor in regulating branching. Light also influences sugar accumulation and transport (Girault et al., 2010; Henry et al., 2011; Rabot et al., 2012). Several studies have suggested that light acts on bud outgrowth by mediating hormones. In rose, prolonged exposure to light led to cytokinin accumulation in the bud by regulating cytokinin synthesis, degradation, and transport and then induced outgrowth (Roman et al., 2016). In Arabidopsis, phyB promoted branching by suppressing auxin signaling (Krishna Reddy and Finlayson, 2014). Genes associated with ABA biosynthesis and signaling could be regulated by R:FR light; thus, ABA regulates bud outgrowth responses to R:FR (Reddy et al., 2013). The altered expression of so many genes involved in the response to light in this study (Dataset S5) indicated that PpEBB1 had a great influence on plants receiving light signals, which may be one of the ways for EBB1 promoting the branching process.

Sugars play major roles during the very early events of bud outgrowth acting as both the source of carbon and signaling molecule (Barbier et al., 2019). Notably, a large number of genes and proteins related to sucrose, glucose, fructose, and starch metabolism were differentially expressed (Figures 2A,B; Datasets S6 and S7) with the overexpression of *EBB1* in this study. As a result, the contents of sucrose, glucose, fructose, and starch were all increased in *PpEBB1-oe* poplar (Figure 2C). A previous study (Tarancon et al., 2017) suggested that carbon starvation might be partly responsible for the transition from active growth to dormancy, which was conserved in woody and herbaceous species.

The positive function of soluble sugars, especially sucrose, on bud outgrowth has also been well studied (Mason et al., 2014; Barbier et al., 2015a). The role of sucrose in promoting bud outgrowth has been studied in many species; for example, the content of sucrose had a strong association with bud outgrowth in wheat (Kebrom et al., 2012), pea (Mason et al., 2014), and sorghum (Kebrom and Mullet, 2015). Sucrose is an early modulator of the key hormonal mechanisms controlling bud outgrowth including auxin and cytokinin by regulating related genes, such as *TAR1*, *YUC1*, *PIN1*, and *IPT3*, in rose under a favorable light environment (Barbier et al., 2015a,b), and the altered expression of these genes was also detected in a previous study (Zhao et al., 2020). Exogenous sucrose could trigger trehalose-6-phosphate (Tre6P) levels in axillary buds of garden pea (*Pisum sativum*), which increased after decapitation, coinciding with the initiation of bud outgrowth (Fichtner et al., 2017). Some crucial genes and proteins associated with Tre6P showed altered expression in this study (Figures 2A, B; Datasets S6 and S7), which was consistent with the study on sorghum that expression of several *TPS* and *TPP* genes were changed in phyB-regulated shoot branching (Kebrom and Mullet, 2016). In rose, sugar promotes bud break accompanied by the import of sucrose (Henry et al., 2011). Sugars rapidly become redistributed and accumulate in axillary buds to trigger bud release after decapitation, and this process may be the initial regulator of apical dominance rather than auxin (Mason et al., 2014). A study on rose (Roman et al., 2016) showed that photosynthesis and sugars play important roles in enhancing growth following bud outgrowth but were not essential for the initial steps of outgrowth, indicating that *PpEBB1* might also take part in subsequent growth after bud break rather than only promoting the initiation of bud break.

Brassinosteroid was also identified as a positive regulator of shoot branching. In this study, overexpression of *EBB1* influenced the expression of biosynthesis of BR, metabolism, and signaling-related genes, which was also supported by a previous study (Yordanov et al., 2014). Study on Arabidopsis found that shoot induction was inhibited in the biosynthesis of BR mutant *dwarf7-1*, while the expression level of *ESR2* was lower, indicating that BR controlling shoot induction was done by regulating *ESR2* (Cheon et al., 2010). A direct connection was found in Arabidopsis so that DRN and DRNL could interact with BES-INTERACTING MYC-LIKE PROTEIN 1 (BIM1), which functions in BR signaling (Chandler et al., 2009). These results further suggested an interaction between PpEBB1 and BRs, but it was unclear which factor was upstream during shoot induction, and there might be a complex relationship between EBB1 and BR. Two *BES1/BZR1* genes, playing important roles in the downstream BR signaling pathway, were upregulated in *PpEBB1-oe* poplar, and they also play critical roles in strigolactone (SL) signaling controlling shoot branching (Hu et al., 2020), providing an important connection between SLs and BRs in regulating shoot branching. Moreover, it has been proposed that auxin, cytokinins, and SL formed a systemic network controlling shoot branching (Domagalska and Leyser, 2011). A previous study has confirmed that overexpression of *PpEBB1* led to a large number of DEGs associated with auxin and cytokinins biosynthesis, transport, and signaling; hence, the increased content of auxin and *tZ*-type cytokinin (the most effective natural cytokinin) were observed (Zhao et al., 2020). Cytokinins are the only hormones known to promote bud outgrowth to date (Domagalska and Leyser, 2011), suggesting cytokinins may work in this PpEBB1-induced shoot branching. SLs were identified to inhibit bud outgrowth and function as a negative regulator of shoot branching (Hu et al., 2020). From the transcriptome data, the *SUPPRESSOR OF MAX2 1-LIKE* (*SMXL*) gene (*Potri.008G069100*), a conversed SL signaling gene, was downregulated in *PpEBB1-oe* poplar (Supplementary Figure S6), suggesting that EBB1 might also influence SL signaling to regulate shoot branching. Auxin was considered as an inhibitor of shoot branching so that auxin export from the bud is crucial for bud activation, but it was also proposed that both decreased and increased auxin transport were associated with increased branching (Domagalska and Leyser, 2011), and auxin export from the axillary bud is not necessary for initiating bud outgrowth (Barbier et al., 2019); hence, the role of auxin in branching is still unclear. Therefore, the integration of various plant hormones including auxin, cytokinins, BR, and SLs play more important roles than acting by themselves individually. Besides being involved in regulating shoot branching, hormones also play important roles in leaf development (Gonzalez et al., 2012), which may be responsible for the changed leaf phenotype in this study.

Nitrogen has profound effects on the formation of the coordinated architecture of both root and shoot systems, and some studies have found that nitrogen participates in the regulation of shoot branching, although the mechanism is not very clear (de Jong et al., 2014). Sufficient nitrogen supply led to more branches with more elongation than limited nitrogen supply in Arabidopsis. In addition, auxin and SL signaling are at least partially required for this process (de Jong et al., 2014). The study on rice (*Oryza sativa* L.) found that nitrogen availability interacted with auxin, cytokinin, and SL to affect shoot branching (Xu et al., 2015). In poplar, high nitrogen positively influences the number of sylleptic branches (Novaes et al., 2009), and cytokinin is likely to participate in this process by mediating long-distance nitrogen signaling (Cline et al., 2006). As one of the products of nitrogen metabolism, amino acids were likely to participate in regulating the bud outgrowth process (Fichtner et al., 2017). In garden pea (*Pisum sativum* L.), decapitation led to the content of most amino acids being significantly increased, which was inconsistent with this study that most amino acids were significantly decreased and only four amino acids were significantly increased (Figure 5). A previous study found that altered expression of *AAP3*, an amino acid transporter, influenced tillering in rice and that RNAi of *AAP3* promoted bud outgrowth, whereas overexpression of *AAP3* increased concentrations of amino acids, which inhibited bud outgrowth and rice tilling (Lu et al., 2018). Lu et al. (2018) also found that amino acid concentrations were critical for plant growth. Most amino acids with low concentrations promoted bud elongation, while enhanced concentrations inhibited bud elongation. Hence, the function of amino acids in regulating shoot branching remains to be further studied. As the precursor of auxin synthesis, the increased Trp content was consistent with the increased level of auxin in *PpEBB1-oe* poplar as observed in a previous study (Zhao et al., 2020). Some individual amino acids have been found to be involved in shoot branchings, such as Asp and glutamic acid (Glu). However, Asp and Glu treatment gave no significant promotion of lateral bud outgrowth in hybrid poplar (Cline et al., 2006). A study on *Rosa hybrida* found that Asp was required to sustain an efficient elongation of the secondary axis after bud outgrowth in combination with sucrose (Le Moigne et al., 2018). Glu is essential for the induction of cytokinin biosynthesis-related genes, such as *adenosine phosphate-iso-pentenyltransferase* (*IPT*) *4* and *IPT5* (Kamada-Nobusada et al., 2013). These results suggested that the function of Asp and Glu on promoting bud outgrowth needs to be combined with other factors. Previous studies (Ohashi et al., 2017, 2018a) found that lack of cytosolic glutamine synthetase1;2 (GS1;2) led to a remarkable reduction in Glu and deficiency in active cytokinin, accompanied by reduced tillers in rice. Moreover, *gs1;2* mutants resulted in decreased sucrose in rice by downregulating *cytosolic fructose 1,6-bisphosphatase2* (*cFBP2*), and *cFBP2* could be upregulated by NH_4_^+^ supply in WT but not in *gs1;2* mutants (Ohashi et al., 2018b). Combined with this study of the altered expression of GS (Figure 4) and FBP (Figure 2), the roles of PpEBB1 in nitrogen metabolism might be an upstream of sugar and hormone metabolism-regulating shoot branching.

Taken together, besides the conserved function in promoting bud-break, EBB1 has a positive role in regulating shoot branching. A large number of DEGs and DEPs in *PpEBB1-oe* poplar are associated with light response, phytohormones, sugars, and nitrogen metabolism; these processes have been considered to participate in regulating shoot branching, suggesting that PpEBB1 may promote shoot branching by coordinating these processes. In addition, the AP2 ERF domain binds to a GCC box (Chandler, 2018). As shown in a previous study, PpEBB1 could directly bind to the GCC box-like element of auxin biosynthesis-related genes including *STY1*, *SRS5*, and *YUC1*, and promote their expression (Zhao et al., 2021). Therefore, some of the DEGs involved in these processes may directly be regulated by PpEBB1 but needs to be further studied.

## Data Availability Statement

The datasets presented in this study can be found in online repositories. The names of the repository/repositories and accession number(s) can be found at: https://www.ncbi.nlm.nih.gov/bioproject, 725066; https://www.ebi.ac.uk/pride/archive, PXD025636.

## Author Contributions

XZ, XC, LL, and XF designed the study. XZ, BW, LL, QT, and CL performed the experiments. XZ wrote the manuscript. All authors contributed to the article and approved the submitted version.

### Conflict of Interest

The authors declare that the research was conducted in the absence of any commercial or financial relationships that could be construed as a potential conflict of interest.

## Footnotes

^1^http://genome.jgi.doe.gov/Ptrichocarpa/download/_JAMO/587b0ae47ded5e4229d885bd/Ptrichocarpa_444_v3.1.gene_exons.gff3.gz?requestTime=1501142324

## Supplementary Material

Dataset S1 Significantly-enriched GO terms of DEGs between *PpEBB1-oe* and WT poplars mentioned in this study.

Dataset S2 Significantly-enriched KEGG pathways of DEGs between *PpEBB1-oe* and WT poplars.

Dataset S3 Significantly-enriched GO terms of DEPs between *PpEBB1-oe* and WT poplars mentioned in this study.

Dataset S4 Significantly enriched KEGG pathways of DEPs between *PpEBB1-oe* and WT poplars mentioned in this study.

Dataset S5 DEGs related to light response.

Dataset S6 DEGs related to sugar metabolism.

Dataset S7 DEPs related to sugar metabolism.

Dataset S8 DEGs related to brassinosteroid (BR) biosynthesis, metabolism, and signaling.

Dataset S9 DEPs related to nitrogen metabolism.
