# Supplementary material for: Overexpression of the Peach Transcription Factor Early Bud-Break 1 Leads to More Branches in Poplar
Source: Front Plant Sci. 2021 Jun 17;12:681283. doi: 10.3389/fpls.2021.681283 (PMC8247907; doi:10.3389/fpls.2021.681283)
Supplement: Supplementary file 1 [file Data_Sheet_1.PDF]

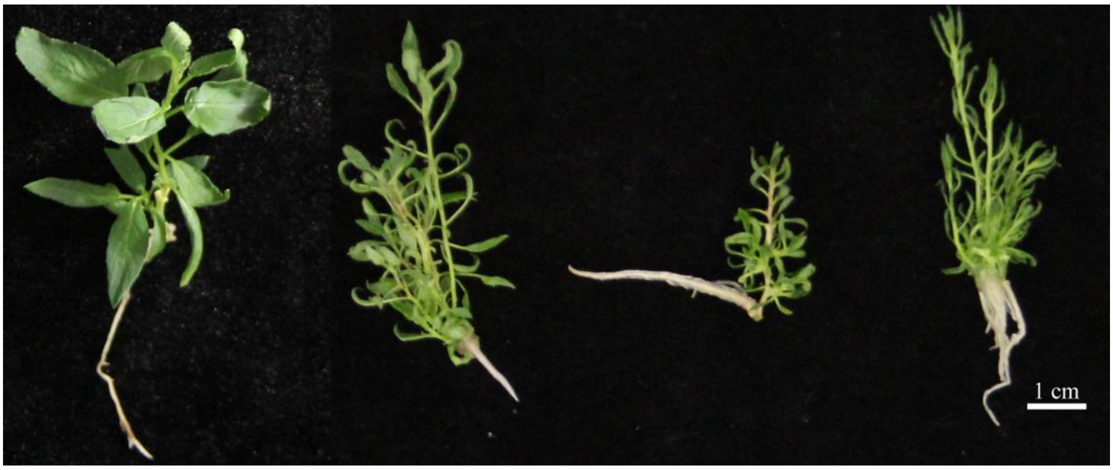

Figure S1. Branches of *PpEBB1-oe* poplars are grown out in subculture.

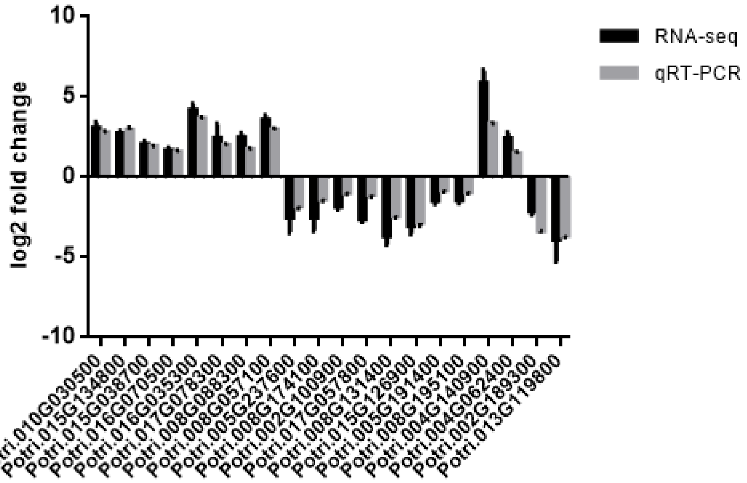

Figure S2. Verification of RNA-seq results via qRT-PCR.

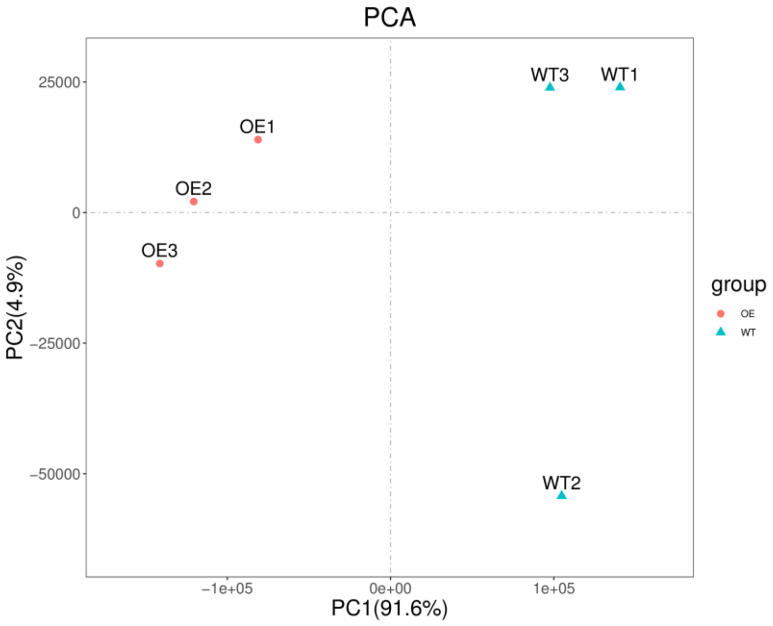

Figure S3. Verification of RNA-seq results via PCA.

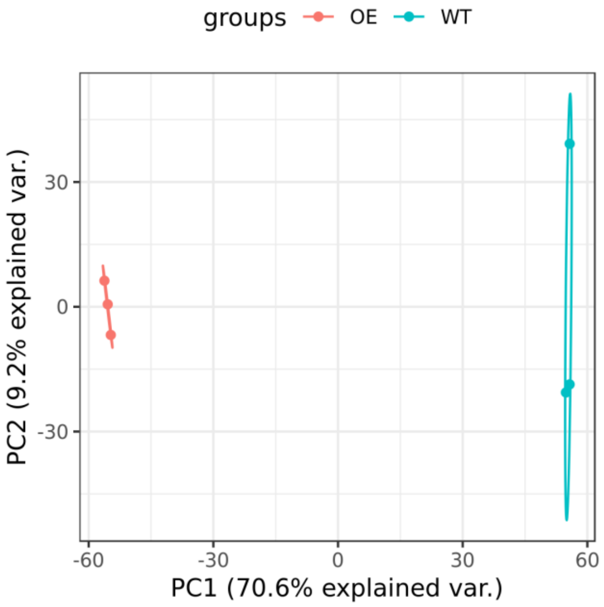

Figure S4. Verification of proteomics results via PCA.

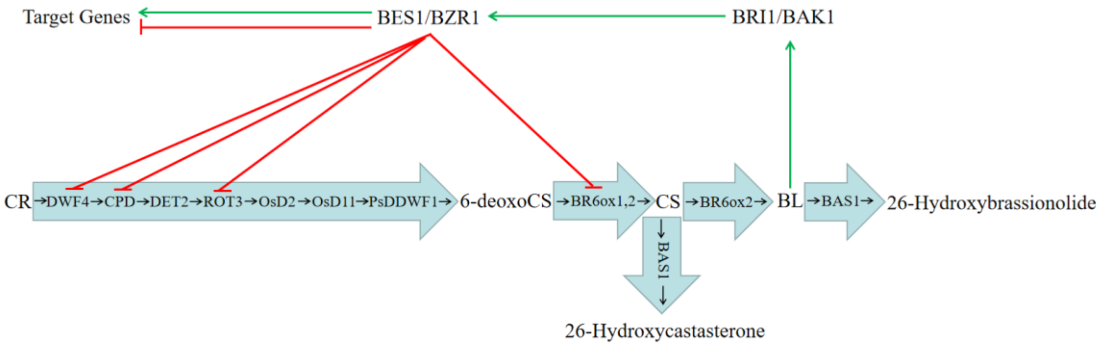

Figure S5. Simplified brassinosteroid biosynthetic pathway according to Zhao and Li (2012).

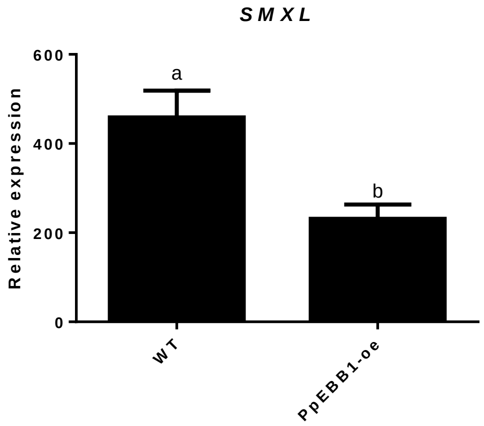

Figure S6. *SMXL* was downregulated in *PpEBB1-oe* poplar.

Table S1. Primers used for qRT-PCR in this study.

Gene name	Forward primer	Reverse primer
Potri.010G030500	CATTCGTATCAGAGCGGGT	CACAATCGCCTCATTTCG
Potri.016G035300	TTATGGCAATGACCTCGG	CAGCACTCTTTGGGTGTAGC
Potri.017G078300	GTCAACATTGCCCAACACT	CTGAAGATGGTGCCACAGT
Potri.015G038700	CAAGGTTCCACCATTACCA	AGGTCCCTACCATCTTCAGC
Potri.008G088300	TGCTACGAGATTAGGTGCG	GGGCAATGTGTTGGAAGA
Potri.015G134800	CAAAGGAAGACGCAACTGA	GGGTCCATCATAATCTGCC
Potri.008G057100	TCACAATAAACGGACACCG	ACCCTGAAAGACAATGACTGAC
Potri.016G070500	GCTGAACTGGCTGGACATT	TCCTTTCCCTCTGCTTCCT
Potri.005G237600	GCTGAAATGGCTCGTCAA	GCTGGTGTGATGAATCCTTC
Potri.008G174100	ATGATGGATGTCTGGTGGG	CTTTGGCAATGGAACCGT
Potri.002G100900	CAGTATCGGCTCTTCTCGTG	TGAGGTGCTTCTTCCTGCT
Potri.017G057800	TCACTTGATGCTGCTGAACT	TGCCAGGAGGAATAACCA
Potri.008G131400	CCTTTGGCTCGCTACACTTA	GGGTTTCCTTCCTTGTCAAC
Potri.015G126900	TCGCCGAATCTATGGTTATC	TAAGAAGCCAAGTGCGGT
Potri.005G191400	CAATGGGAAACTGCGTGT	TTCTTCTCTTCAGCGGCA
Potri.008G195100	CATCTTGCTCGTGCCTACA	CACATCCTCAATCTCCTTGC
Potri.004G140900	CAAGATTTGTGCTGGTGACT	ACGGATTCCATACTGGGA
Potri.004G062400	CACACTGGTTCCATAAGCG	GGTAGACGAGGGCATAGATG
Potri.002G189300	GCCAGGAATGGAGTCAAGTT	CAATGCGGTAACCCTCAAC
Potri.013G119800	AATACATCATCTGGCGGC	ATTTGTGTCCTCCAAGTCCT
PtActin	GATTTGTCCCTCGCGCTGT	TCGGTATAATGACCCTTGGCC
